# Multi‐Wavelength Achromatic 3D Meta‐holography with Zoom Function

**DOI:** 10.1002/advs.202501881

**Published:** 2025-04-25

**Authors:** Chao Liu, Yi Zheng, Di Wang, Qian Huang, Xiao‐Wei Li, Fan‐Chuan Lin, You‐Ran Zhao, Yi‐Wei Zheng, Xiao‐Ke Lu, Xin‐Ru Li, Xin‐Ru Zheng, Xin Xie, Kun Song, Zhen‐Fei Li, Wei Lu, Din Ping Tsai, Ruo‐Nan Ji, Qiong‐Hua Wang

**Affiliations:** ^1^ School of Instrumentation and Optoelectronic Engineering Beihang University Beijing 100191 China; ^2^ State Key Laboratory of Infrared Physics Shanghai Institute of Technical Physics Chinese Academy of Sciences Shanghai 200083 China; ^3^ School of Physical Science and Technology Northwestern Polytechnical University Xi'an 710129 China; ^4^ Department of Electrical Engineering City University of Hong Kong Hong Kong 999077 China

**Keywords:** metasurface, multi‐wavelength, liquid lens, meta‐holography

## Abstract

With the development of nanofabrication technology, meta‐holography has shown unprecedented potential for light regulation. However, due to the wavelength mismatch, chromatic aberrations are inevitable in multi‐wavelength meta‐holography. Moreover, since the meta‐hologram is difficult to refresh, zoom meta‐holography has not been reported to date. Here, a multi‐wavelength achromatic 3D meta‐holography with a zoom function is developed, breaking through both the chromatic aberration interference and imaging limit for the first time. A wide‐band 3D meta‐hologram is generated and is used to design a wide‐band metasurface fabricated with nanostructures based on the Pancharatnam–Berry phase, thus achieving the multi‐wavelength meta‐holography. A fast‐tunable liquid lens with a large zoom range is designed, where a high elasticity polymer membrane and a high refractive index composite liquid with suitable viscosity and density are prepared to overcome the bottlenecks of limited zoom range and response speed. By jointly controlling the two liquid lenses, multi‐wavelength achromatic 3D meta‐holographic images with adjustable depth and size are successfully reconstructed. The zoom ratio of the meta‐holographic images can reach 2.1, and the zoom response time can reach 5 ms. Such a tunable multi‐wavelength achromatic 3D meta‐holography has broad applications in storage, display, and information security.

## Graphical abstract

Due to the wavelength mismatch, chromatic aberrations are inevitable in multi‐wavelength meta‐holography. Moreover, since the meta‐hologram is difficult to be refreshed, zoom meta‐holography has not been reported to date. This study proposes a multi‐wavelength achromatic 3D meta‐holography with a zoom function, breaking through both the chromatic aberration interference and imaging limit in meta‐holography.

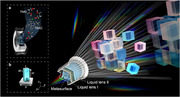

## 1 Introduction

Holography can accurately and completely recover the wavefront information of 3D objects, which has important applications in frontier fields such as encryption, microscopic imaging, and beam shaping.^[^ ^1^, ^2^, ^3^ ^]^ 3D holography with full color, large viewing angle, and adjustable size is a core development direction in the field of holography.^[^ ^4^, ^5^, ^6^, ^7^ ^]^ However, traditional holography modulates the light field based on micron‐sized refreshable devices such as spatial light modulator (SLM), which limits the viewing angle to be less than 9° even using the most advanced SLM (pixel size is 3.6 µm), thus seriously hindering its applications.^[^ ^8^, ^9^, ^10^ ^]^ With the development of nanofabrication technology, meta‐holography has shown unprecedented potential for light modulation.^[^ ^11^, ^12^, ^13^, ^14^, ^15^ ^]^ With the sub‐wavelength pixel pitch, meta‐holography can easily achieve a wide viewing angle of more than 22° in a compact system, which has attracted great attention.^[^ ^16^, ^17^, ^18^ ^]^

Although meta‐holography provides sufficient viewing angle, the challenge of realizing multi‐wavelength 3D meta‐holography with zoom function has not been overcome.^[^ ^19^, ^20^, ^21^ ^]^ Multi‐wavelength 3D meta‐holography with zoom function can not only greatly increase the information capacity, but also be very important for accurately identifying the detailed information of objects.^[^ ^22^ ^]^ For example, in the fields of detection and microscopic imaging, large information capacity and adaptive size and depth adjustments are critical to the results.^[^ ^23^ ^]^ However, due to wavelength mismatch, chromatic aberrations are inevitable in color meta‐holography. Some researchers integrated wavelength‐dependent cell units into a metasurface to realize color meta‐holography.^[^ ^24^, ^25^, ^26^ ^]^ Some researchers designed an angle‐dependent metasurface, which can produce different phase modulation by controlling the angle of incident light, thus providing a potential solution for color meta‐holography.^[^ ^27^ ^]^ In addition, the polarization multiplexing method is also widely used in meta‐holography.^[^ ^28^, ^29^, ^30^, ^31^, ^32^ ^]^ By introducing polarization information, the decoupling problem in wavelength dimension can be solved. However, the existing color meta‐holographic methods usually sacrifice polarization information or spatial dimension information to eliminate chromatic aberration,^[^ ^33^, ^34^, ^35^ ^]^ and the reconstructed 3D depth is limited by spatial sampling frequency and pixel pitch.^[^ ^36^ ^]^ More seriously, traditional methods only enable chromatic aberration compensation for specific wavelengths, causing limitations in dynamic chromatic aberration compensation of meta‐holography with any wavelengths.

Multi‐wavelength 3D meta‐holography with zoom function can not only greatly increase the information capacity, but also adaptively adjust the imaging size and depth, which has important applications in fields such as optical storage and encryption.^[^ ^37^, ^38^, ^39^ ^]^ In multi‐wavelength meta‐holography, it is necessary to consider the response of meta‐holograms to different wavelengths, which poses new challenges to the coding of holograms and the design of metasurfaces. Moreover, since the meta‐hologram cannot be refreshed, zoom meta‐holography has not been reported so far.

**Figure 1:**
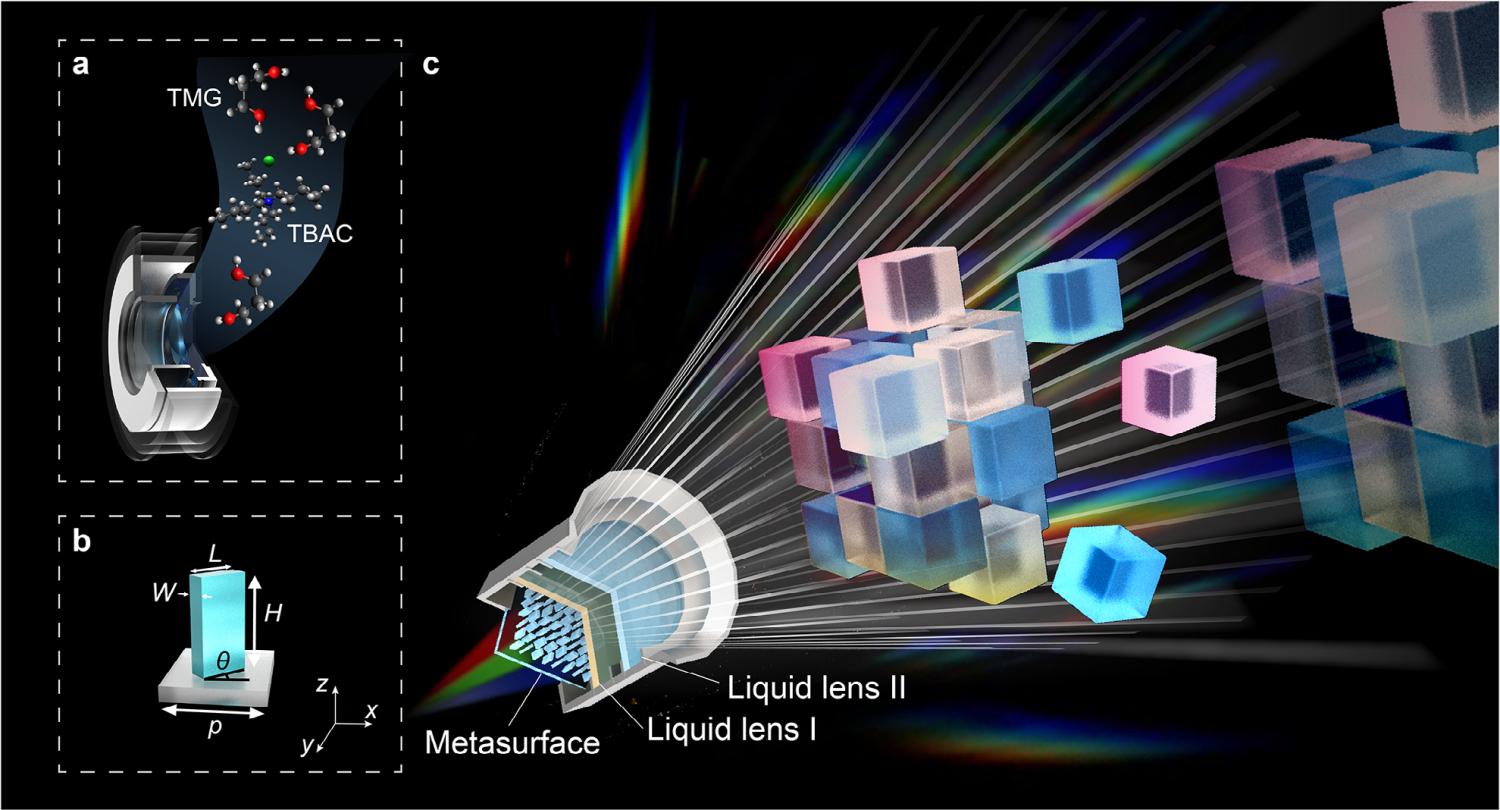
Concept of the multi‐wavelength achromatic 3D meta‐holography with zoom function. a) Schematic diagram of the liquid lens. b) Schematic diagram of the unit cell of the metasurface. c) Schematic diagram of the multi‐wavelength achromatic 3D meta‐holography with zoom function.

Here, a multi‐wavelength achromatic 3D meta‐holography with a zoom function based on liquid lenses is proposed to break through the chromatic aberration interference and imaging limit, as shown in **Figure** **1**. This strategy represents a great advance in meta‐holography for the following reasons. First, this approach is effective in realizing multi‐wavelength 3D meta‐holography. A metasurface is prepared based on the wide‐band nanostructures. When three colors of light illuminate the metasurface, color meta‐holographic 3D images can be reconstructed. The proposed method does not need to separately calculate the meta‐holograms with different wavelengths. Second, the proposed meta‐holography solves the difficulties in realizing achromatic reconstruction and zoom function. A fast tunable liquid lens with a large zoom range is designed to adjust the imaging size and depth. To overcome the bottlenecks of limited zoom range and response speed in liquid lenses, a high elasticity polymer membrane and a composite liquid composed of tetrabutylammonium chloride (TBAC) and propane‐1,3‐diol (TMG) are developed. Based on the visual persistence effect of human eyes, the liquid lenses are used to correct the image size and position in time sequence, then the chromatic aberration is eliminated and the limitation of the imaging depth of meta‐holography is broken. Third, different from the traditional color meta‐holography which can only pre‐compensate chromatic aberration for several specific wavelengths, the proposed method can dynamically compensate the chromatic aberration for any wavelengths. By precisely designing monocrystalline silicon‐based metasurface, and fast tunable and large zoom range liquid lenses, the shortest zoom response time can reach 5 ms, the zoom ratio can reach 2.1, and the imaging depth can be flexibly adjusted within the range above 2.7 cm. The experiments also show the potential of color meta‐holography with a 64° wide viewing angle. Such multi‐wavelength and zoom 3D meta‐holography is expected to open a new avenue for storage, display, and information security.

## 2 Results

The realization of the proposed multi‐wavelength achromatic 3D meta‐holography with zoom function depends on the design of the high‐resolution wide‐band meta‐hologram and the fabrication of fast tunable and large zoom range liquid lenses. A wide‐band 3D meta‐hologram is generated first. On this basis, a metasurface with a wide‐band response based on the Pancharatnam‐Berry (PB) geometric phase is designed, so that 3D meta‐holographic images with different wavelengths can be successfully reconstructed. To eliminate the chromatic aberration in color images and adjust the imaging size and depth, a fast‐tunable liquid lens with a large zoom range based on new materials is proposed.

### 2.1 Mechanism of Chromatic Aberration Compensation and Zoom

**Figure 2:**
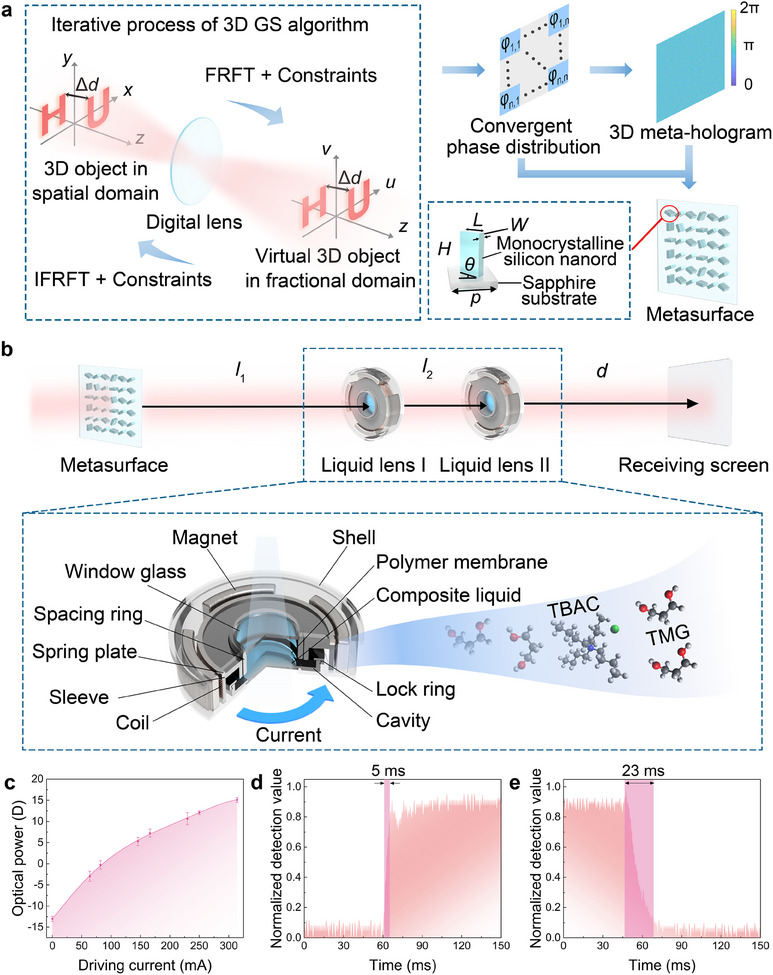
Principle of the multi‐wavelength achromatic 3D meta‐holography with zoom function. a) Computation strategy of the meta‐hologram. b) Analysis of chromatic aberration compensation and structure of the proposed liquid lens. c) Optical power range of the proposed liquid lens. d) Rise response time and e) fall response time of the proposed liquid lens.

A wide‐band 3D meta‐hologram recording the image information of a 3D object consisting of multiple patterns in different planes is generated for multi‐wavelength reconstruction, as shown in **Figure** **2**a (Section S1, Supporting Information). The size of the reconstructed image can be expressed as (taking the *y* direction as an example):

(1) $$L_{y}=\frac{m^{\prime }\lambda f\sin^{2}\left(a\pi /2\right)}{mdu}$$

where *L_y_* represents the length of the reconstructed image in the *y* direction, *du* represents the pixel pitch of the meta‐hologram along *y* direction, *λ* represents the wavelength of the incident light, *f* is the focal length of the Fourier lens, *a* is the transformation order, *m* and *m'* represent the number of pixels of the meta‐hologram and the target object along *y* direction, respectively. On this basis, the size of the reconstructed images differs with the wavelength, causing chromatic aberration.

In order to compensate for the chromatic aberration, two liquid lenses are used to adjust the sizes and depths of the meta‐holographic images, as shown in Figure 2b. The distance from the metasurface to liquid lens I is set to *l*_1_, the distance between the two liquid lenses is set to *l*_2_, and the distance from liquid lens II to the receiving screen is set to *d*. When the receiving screen is located at the focal plane, according to the Gaussian formula, it can be derived that:

(2) $$\frac{1}{d}+\frac{1}{l_{2}-f_{1}}=\frac{1}{f_{2}}$$

where *f*_1_ and *f*_2_ are the focal lengths of the two liquid lenses, respectively. For the proposed meta‐holography with zoom function, the positions of the metasurface, lens I, and lens II are all fixed, which does not require mechanical movement of any component.

The distance *d* between the receiving screen and the lens II can be defined as the imaging depth for the image with a transformation order of 1.00:

(3) $$d=\frac{f_{2}\left(l_{2}-f_{1}\right)}{l_{2}-f_{1}-f_{2}}$$

When the receiving screen is fixed, the image size can be expressed as (taking the *y* direction as an example):

(4) $$L_{y}=\frac{m^{\prime }f_{1}\lambda d}{m\left(f_{1}-l_{2}\right)du}$$

According to Equations (3) and (4), the focal lengths of the liquid lenses required to enable holographic images of different wavelengths to be imaged at the same depth and with consistent size can be calculated. By adjusting *f*_1_ and *f*_2_, the chromatic aberration can be well compensated through time‐division multiplexing. Additionally, the imaging depth and image size can be also flexibly adjusted, achieving a zoom function. The achromatic zoom range of the meta‐holographic image is determined by the zoom lens group consisting of the two liquid lenses and the adopted spectral band (Section S2, Supporting Information).

### 2.2 Design of the Liquid Lens

To achieve flexible adjustment of the imaging size and depth of multi‐wavelength meta‐holography, a fast‐tunable liquid lens with a large zoom range is designed. A polymer membrane with high elasticity and transmittance is first prepared. Considering that polymer material may undergo hydrolysis, swelling, and permeation in many types of liquids, we select transparent alcohol category substances with strong polarity as the basic liquid to ensure the physicochemical stability between the polymer membrane and the liquid. Moreover, in addition to thermal stability and low volatility, the selected liquid should also have high refractive index, moderate viscosity, and low density similar to the polymer membrane, which can be beneficial to improving the zoom range, response speed, and anti‐gravity interference capability of the liquid lens.

Single alcohol category substances are difficult to meet all the above property requirements. Through analysis of multicomponent physicochemical properties, we propose a composite liquid, consisting of an appropriate amount of TBAC as the solute, and TMG as the solvent (Section S3, Supporting Information). Different from many traditional aqueous salt solutions where both the refractive index and density are positively correlated, it is found that adding TBAC to TMG not only increases the refractive index, but also reduces the density of the liquid, which helps to prepare a relatively ideal liquid for the proposed liquid lens. Additionally, due to the appropriate internal friction between molecules, the viscosity of the prepared liquid is always within the appropriate range when adding TBAC, within which the response speed of the liquid lens is relatively fast without introducing violent oscillations.

In terms of driving mode, we design a voice coil motor actuator structure, which provides a rapidly generated controllable electromagnetic force for the single‐phase liquid filled with the polymer membrane, and the motion of the fluid is accelerated under the action of forced pressure, thereby shortening the response time. By controlling the driving current in the coil, the actuator will generate axial displacement, and the compression force on the polymer membrane and composite liquid can be changed, causing changes in the distribution of hydraulic pressure inside the cavity and deformation of the optical interface, thus realizing zoom function. The developed liquid lens effectively meets the requirements of meta‐holography, including a large zoom range, fast response speed, and good imaging performance. Wide band and polarization‐independent properties of the liquid lens also enable chromatic aberration compensation and lossless modulation of the meta‐holograms within the spectral band.

In terms of material preparation of the liquid lens, the prepared polymer membrane utilizes polydimethylsiloxane as the base material, which has a high tensile strength of ≈6.0 Mpa, excellent elastic deformation ability, and high transmittance of over 95%. The composite liquid consists of TBAC and TMG as the solute and solvent, respectively, and the mass fraction is 7.0%. It has a high refractive index of 1.4407, and a low density of 1.044g cm^−3^ similar to the polymer membrane. The viscosity is 44.7 cP, which is within a relatively ideal range, ensuring a fast response speed without introducing violent oscillations. The detailed manufacturing procedure of the liquid lens can be referred to Section S4 (Supporting Information).

### 2.3 Experimental Tests of the Liquid Lens

To verify the advantages of the response time and the zoom range of our proposed liquid lens, experiments are conducted. Results indicate that the maximum range of the optical power (the reciprocal of focal length) of the liquid lens covers around ‐13 D–15 D, as shown in **Figure** **2**c. The sufficiently large zoom range and suitable distance between the two liquid lenses enable flexible adjustment of the depth and size of multi‐wavelength meta‐holographic images within a wide range. In addition, the response time of the liquid lens is also tested, as shown in Figures 2d,e. A photodetector is used to record the illumination change of the light beam passing through the liquid lens, so as to track the dynamic zoom process of the liquid lens. The rise response time is defined as the duration time required for the illuminance detection value to increase from 10% to 90% of the peak value when the driving current is applied to the liquid lens, which is tested at ≈5 ms. The fall response time is defined as the duration time required for the illuminance detection value to decrease from 90% to 10% of the peak value after the driving current is removed, which is tested as ≈23 ms.

Experimental results indicate that our proposed method has an extremely fast zoom speed and can achieve excellent chromatic aberration compensation characteristics through timing control. The fluctuations at the beginning and end of the response curve are mainly caused by the measurement noise, not the unstable optical power of the liquid lens (Section S4, Supporting Information). In addition, a multi‐channel liquid lens driver is also developed to provide synchronous control driving currents for the two liquid lenses (Section S5, Supporting Information).

**Figure 3:**
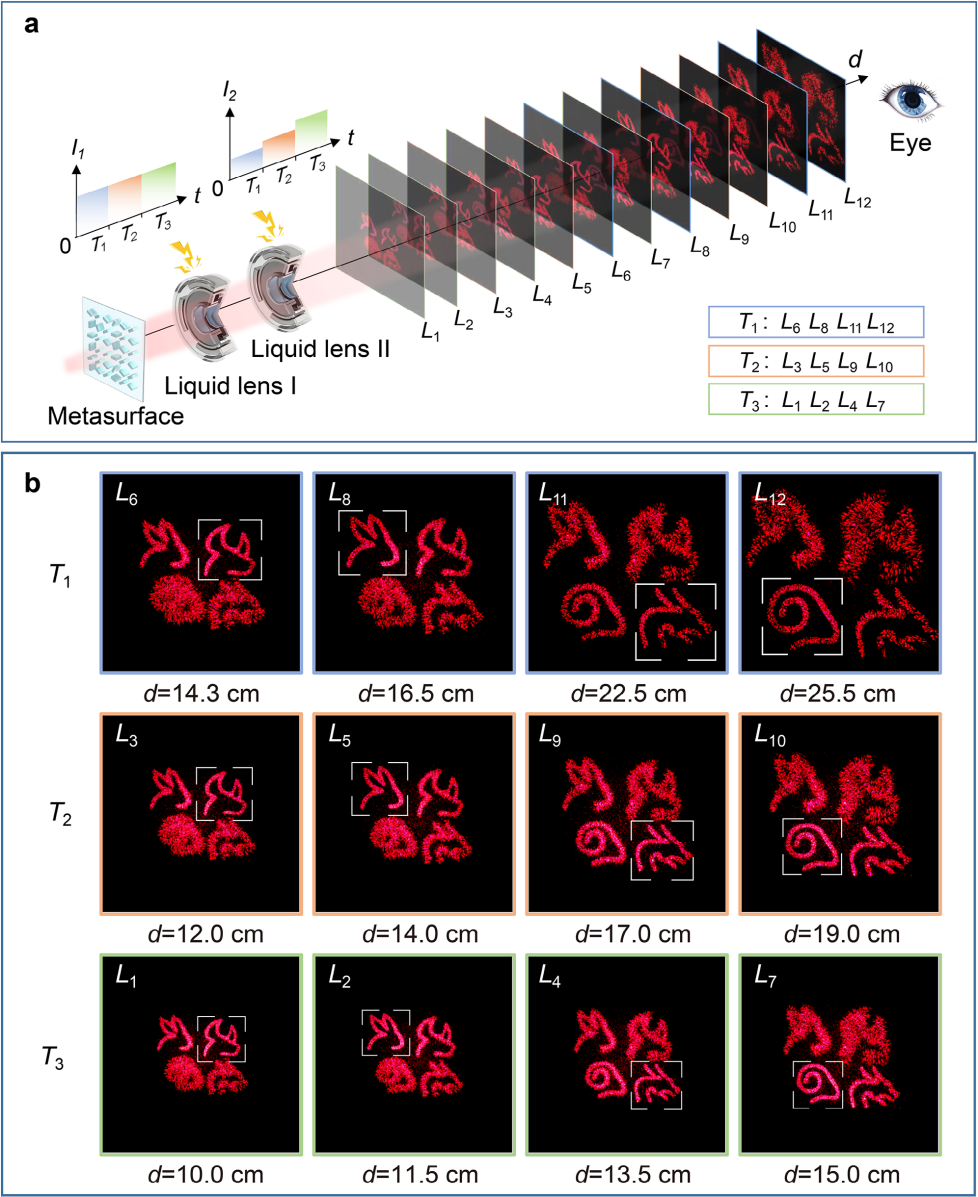
Demonstration of the zoom 3D meta‐holographic display. a) Schematic diagram of zoom 3D meta‐holographic display based on the proposed 3D meta‐holography with zoom function. b) Dynamic zoom 3D holographic display results under the dynamic adjustment of the liquid lenses. (The regions within the white boxes in the images represent the focused parts.)

Then, to verify the feasibility of the zoom modulation function of liquid lenses on meta‐holographic images, four patterns of “ox,” “rabbit,” “dragon,” and “goat” in different planes are selected as the 3D display target. The fractional transformation orders of “ox,” “rabbit,” “dragon” and “goat” are set to 0.90, 0.95, 1.05, and 1.10, respectively in the holographic calculation, which realizes 3D holographic display with four layers. Then, by adjusting the driving currents of the liquid lenses, the depths and sizes of the images can be easily adjusted. **Figure** **3**a shows the process of presenting three different zoom states in sequence when the periodic control signals are applied to the liquid lenses. The currents applied to the two liquid lenses during the duration time *T*_1_, *T*_2_, and *T*_3_ are 123 and 96 mA, 123 and 109 mA, 123 and 122 mA, respectively.

During the visual retention time, three 3D holographic images with different zoom states can be all observed. To provide visualization results, an image sensor is installed on a precision displacement slide rail to capture the images at different depths, as shown in Figure 3b. It can be clearly seen from the experimental results that there are significant changes in the clear imaging positions and sizes of different patterns under the dynamic adjustment of the liquid lenses, proving the effectiveness of zoom 3D meta‐holography by controlling the driving currents of the two liquid lenses.

### 2.4 Design of the Metasurface

To verify the feasibility of the proposed multi‐wavelength achromatic 3D meta‐holography with zoom function, a wide‐band 3D meta‐hologram is generated. A 3D object composed of the letters “H” and “U” in different depth planes is selected as the recorded object for the meta‐hologram. The resolution of the recorded meta‐hologram is 2500 × 2500, and the height of the letters is ≈300 pixels. During the recording process, the focal length *f* is set to 5 cm, and the transformation orders of letters “H” and “U” are set to 1 and 0.6 respectively, which means the 3D depth of the object is recorded as 2.94 cm.

The 3D meta‐hologram is encoded on a transmittive‐type metasurface, which is fabricated on Silicon on Sapphire (SOS) substrates. This metasurface comprises unit cells, each consisting of a rectangular monocrystalline silicon nanorod. To achieve wide‐band non‐dispersive phase control, the PB phase is employed. This is accomplished by rotating the principal axis of the birefringent nanorod, thereby mapping the desired phase profile *φ*(*x*, *y*) onto the metasurface. Specifically, the relationship between the PB phase shift *φ* and the rotation angle *θ* is given by *φ* = ±2*θ*. The positive sign is applicable when the polarization state of the incident light is left circular polarization, whereas the negative sign is used for right circular polarization.

**Figure 4:**
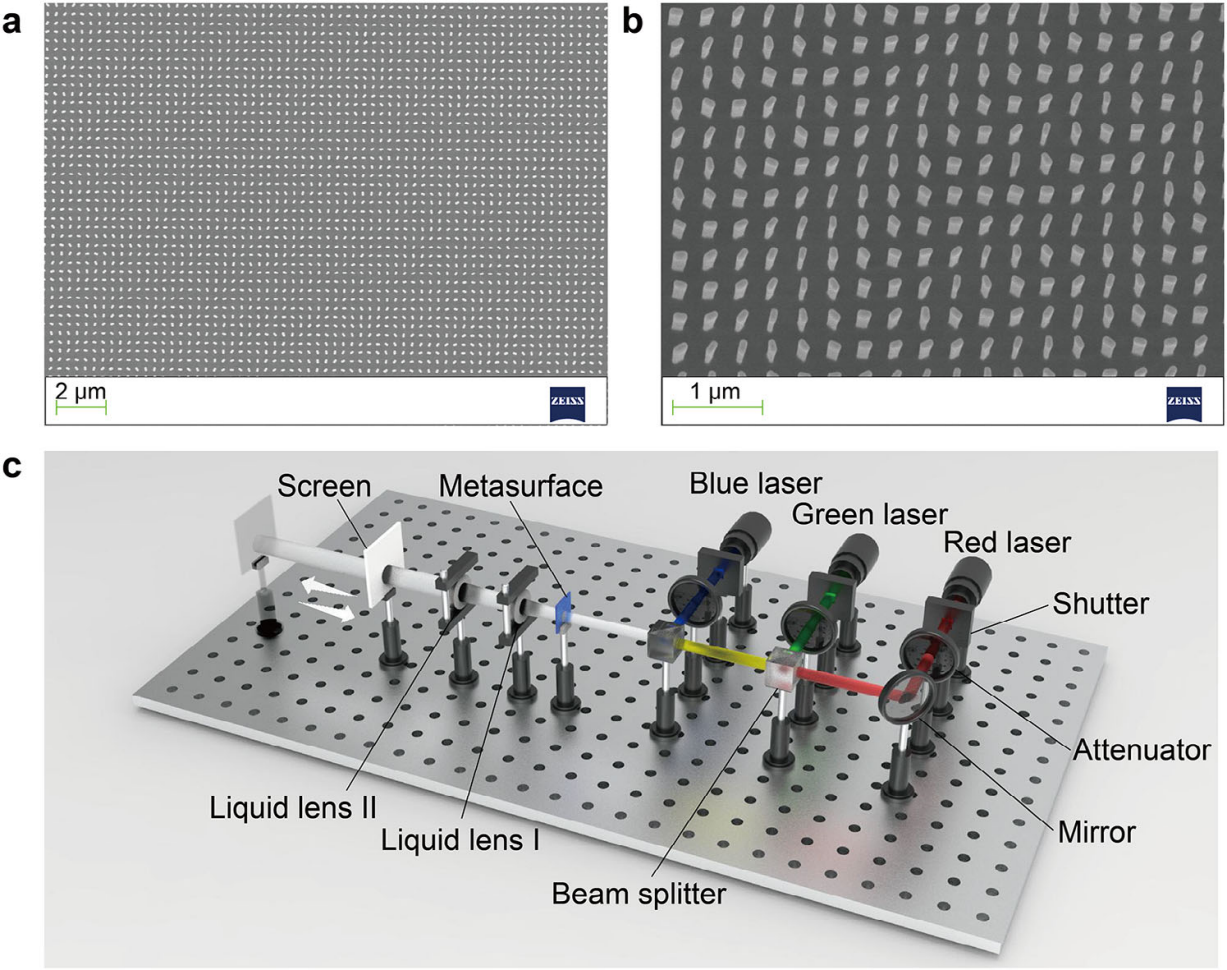
Experimental configuration and realization of 3D meta‐holography. a) Top view and b) side view of the metasurface. c) Schematic diagram of the optical path of the experiment setup.

The metasurface based on PB phase is fabricated accordingly with a pixel pitch of 0.35 µm, and the period (*p*), height (*H*), length (*L*), and width (*W*) of the nanorod are set to 350, 600, 160, and 80 nm, respectively. The scanning electron microscope images of the fabricated metasurface sample are shown in **Figures** **4**a,b. The transmittance of the fabricated metasurface is higher than 26% within the spectral band between 400 nm and 1100 nm, which means that the metasurface has a very wide spectral band (Section S6, Supporting Information).

### 2.5 Meta‐Holographic Reconstruction

The meta‐holographic reconstructed system is shown in **Figure** 4c, which consists of three lasers with different wavelengths, three shutters, three attenuators, a mirror, two beam splitters, a metasurface, two liquid lenses, and a screen. Three lasers with wavelengths of 638, 532, and 473 nm respectively are used as illumination sources. Each laser is connected to a shutter and an attenuator for modulating light intensity. The distance between the two liquid lenses is fixed at ≈3 cm, and the zoom lens group is tightly attached to the metasurface. When the metasurface is illuminated, the focused meta‐holographic images of the letters “H” and “U” can be reconstructed at different depth planes.

**Figure 5:**
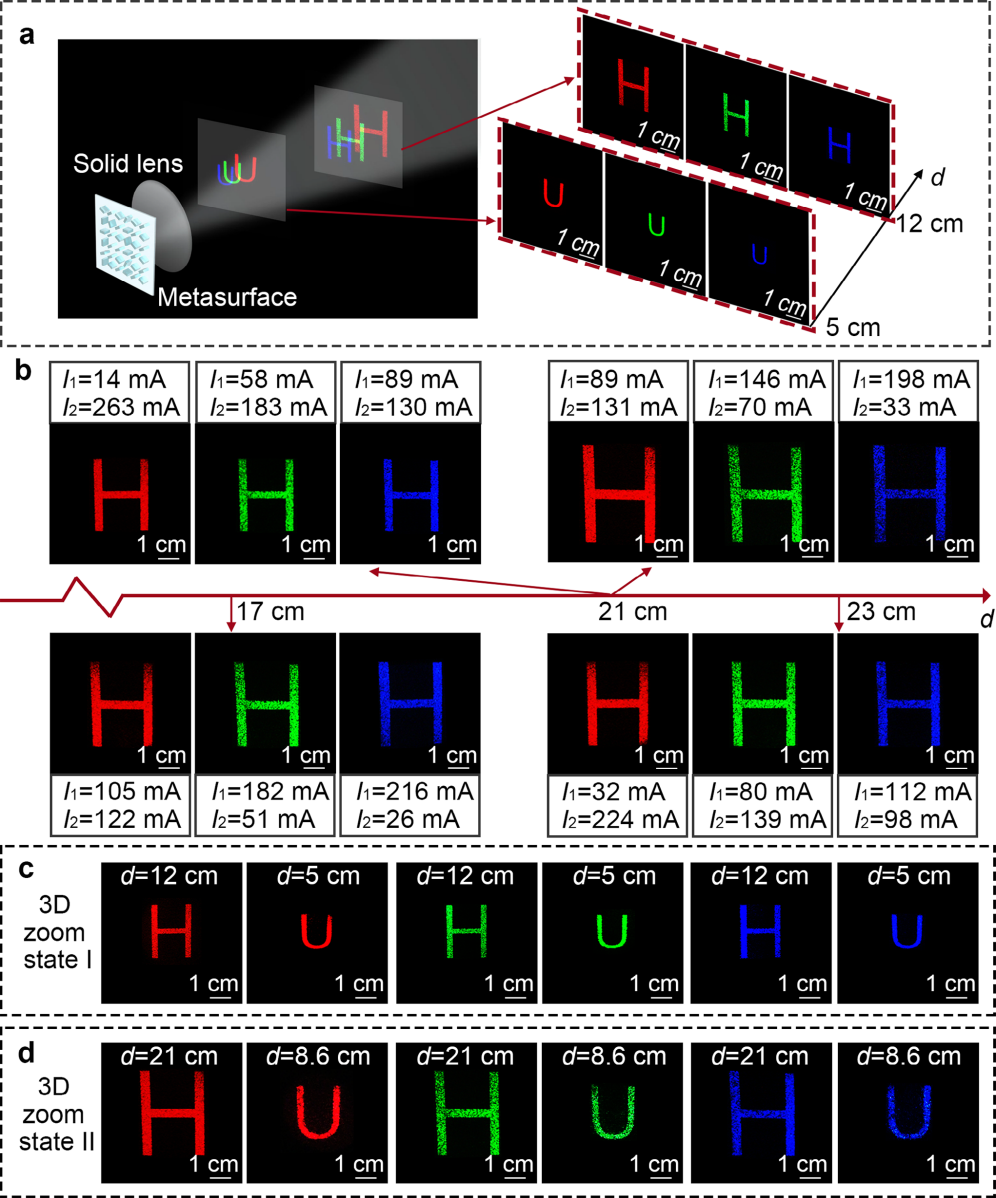
Experiment results of the multi‐wavelength achromatic 3D meta‐holography with zoom function. a) 3D multi‐wavelength meta‐holographic results with chromatic aberration using a solid lens. b) Multi‐wavelength meta‐holographic results with zoom function using the liquid lenses. c,d) Multi‐wavelength achromatic 3D meta‐holographic results with zoom function using the liquid lenses.

We first replace two liquid lenses with a solid achromatic Fourier lens with a fixed focal length of 12 cm. As shown in **Figure** **5**a, the focused meta‐holographic images of the letters “H” and “U” can be captured at depths of ≈12 and 5 cm, respectively. This demonstrates the effectiveness of the proposed method in multi‐wavelength 3D meta‐holographic reconstruction, but a significant chromatic aberration of the three color meta‐holographic images is observed.

To realize the zoom and chromatic aberration compensation functions for multi‐wavelength meta‐holography, liquid lenses are introduced to adjust the size and depth of the meta‐holographic image. The meta‐holographic image of the letter “H” is taken as an example, as shown in Figure 5b. At the same depth, the size of the meta‐holographic image can be adjusted by changing the voltages of the two liquid lenses. Besides, the depth of the meta‐holographic image can also be changed while keeping the image size fixed. The results demonstrate the feasibility of realizing the multi‐wavelength meta‐holographic zoom function. Under the parameters set in the experiment, the size variation range of the color meta‐holographic image of the letter “H” can cover 3.4– 4.6 cm at a depth of 21 cm (Section S7, Supporting Information). It can be calculated that the maximum zoom ratio of the meta‐holographic images with each wavelength can reach ≈2.1, and when both liquid lenses are in the maximum positive optical power state, the minimum projection depth is ≈2.7 cm. Theoretically, the maximum depth can be infinite, but when the diffraction distance is very large, the projection efficiency will be affected.

Furthermore, to demonstrate the ability to realize multi‐wavelength zoom achromatic 3D meta‐holography, the 3D meta‐holographic images consisting of the letters “H” and “U” in different planes are also captured. By changing the driving currents of the liquid lenses, the achromatic meta‐holographic images of the letter “H” with the same size of 2.5 cm can be captured at the depth plane of 12 cm, and the achromatic meta‐holographic images of the letter “U” with the same size of 1.6 cm can be captured at the depth plane of 5 cm, as shown in Figure 5c. Meantime, by further changing the driving currents of the liquid lenses, the achromatic meta‐holographic images of the letter “H” with the same size of 4.0 cm and the achromatic meta‐holographic images of the letter “U” with the same size of 2.6 cm can be captured at the depth planes of 21 and 8.6 cm, respectively, as shown in Figure 5d. The results indicate that the chromatic aberration compensation and zoom function of the 3D meta‐holography are successfully realized through the proposed zooming strategy using liquid lenses.

## 3 Discussion

The above experimental results demonstrate the feasibility of our proposed multi‐wavelength achromatic 3D meta‐holography with zoom function. The proposed method can adaptively adjust the imaging size and position of multi‐wavelength 3D meta‐holographic images, providing a flexible and universal solution for meta‐holography. It should also be noted that our proposed method is not limited to realizing the reconstruction of achromatic 3D meta‐holographic images with the selected three wavelengths. In fact, 3D meta‐holographic images with any wavelengths within the spectral response band of the metasurface and liquid lenses can be modulated by illuminating the metasurface with light of corresponding wavelength and controlling the liquid lenses, and chromatic aberration compensation can be easily achieved (Section S8, Supporting Information). **Table** **1** compares the characteristics between this work and some other state‐of‐the‐art of 3D meta‐holography.

**Table 1:** Comparison of characteristics between this work and some other state‐of‐the‐arts of 3D meta‐holography.

Methods	Wavelengths	Zoom response time	Zoom ratio	Imaging depth
This work	Visible light	5 ms	2.1	≥27 mm
Malek et al.^[^ ^40^ ^]^	632.8 nm	>1 s	1.24	≤0.23 mm
Xiong et al.^[^ ^14^ ^]^	700 nm	No zoom function		≤0.83 mm
So et al.^[^ ^41^ ^]^	635, 532, 450 nm	No zoom function		≤1.5 mm

In addition, our proposed method also shows significant application value in the fields of optical encryption and storage. Due to the zoom capability of our proposed method, the depth and size of each layer of the patterns can be precisely and flexibly adjusted, expanding display channels or layers, thereby enabling a large information capacity and strong information expression ability. More importantly, this method of expanding display channels or layers is not limited by wavelength or polarization selectivity. The designed metasurface can also have the function of polarization adjustment, and if different images are reconstructed by using the polarization characteristics of meta‐holography, the information capacity will be further increased. Therefore, the proposed method demonstrates significant potential to be used in the fields of optical encryption and storage.

Admittedly, achieving an achromatic 3D holographic display containing more wavelength channels in real time remains challenging in its current state. Since the proposed chromatic aberration compensation strategy relies on time‐division multiplexing control of liquid lenses, real‐time multi‐channel chromatic aberration compensation implies higher requirements for the adjusting speed of liquid lenses. But it is indeed feasible by further improving the response speed of the liquid lens through some ways including special design of control signals and optimization of liquid lens components,^[^ ^42^, ^43^ ^]^ which can be our future work.

## 4 Conclusion

In conclusion, we propose a novel multi‐wavelength achromatic 3D meta‐holography with a zoom function, which provides an efficient solution to realize multi‐wavelength achromatic 3D meta‐holography. With the wide‐band nanostructures based on PB phase control, a wide‐band metasurface is fabricated to achieve 3D meta‐holography with any wavelengths within the spectral band. A zoom lens group consisting of two fast tunable and large zoom range liquid lenses is designed to realize chromatic aberration compensation and flexible adjustments of depth and size, which provides a new strategy for achieving zoom color meta‐holography. Furthermore, the proposed method also shows the advantages of greatly increasing the information capacity, which has important applications in fields such as optical storage and encryption.

## 5 Experimental Section

### Metasurface Fabrication

The fabrication of meta‐hologram samples on SOS substrates begins with a thorough substrate cleaning process, which includes sequential immersions in N‐Methyl‐2‐pyrrolidone (NMP), isopropyl alcohol (IPA), and deionized water to ensure surface cleanliness. Subsequently, a 100 nm thick layer of hydrogen silsesquioxane (HSQ) was spin‐coated onto the substrate and then baked at 100 °C for 1 min to solidify the film. Moreover, to counteract electron charging, which can impede the electron beam lithography (EBL) process due to the substrate's poor conductivity, a conductive adhesive layer (AR‐PC 5092) was spin‐coated over the HSQ layer. Subsequently, the EBL process was employed to pattern the HSQ layer, creating the desired nano‐pillow structures. Following this, the pattern was developed by immersing the substrate in a 2.38% NMD‐3 developer solution for 2 min, which selectively removes the exposed HSQ. Ultimately, the final step involves transferring the pattern to the substrate using inductively coupled plasma (ICP) etching. This etching process selectively removed the unprotected silicon, thereby yielding the metasurface with monocrystalline silicon pillars.

## Conflict of Interest

The authors declare no conflict of interest.

## Author Contributions

C.L., W.L., D.P.T., R.N.J., and Q.H.W. conceived the project; C.L., Y.Z., and R.N.J. proposed the idea, performed the simulations and conducted the experiments; X.W.L. and Y.R.Z. fabricated the liquid lens; D.W. and Q.H. proposed and validated the meta‐hologram generation method; X.R.L., X.R.Z., X.X., and K.S. fabricated the metasurface and analyzed the data; F.C.L., Y.W.Z., X.K.L., and F.Z.L. assisted in the verification experiments of meta‐holography. All authors discussed the results and commented on the paper. C.L. and Y.Z. contributed equally to this work.

## Supporting information



Supporting Information

## Data Availability

The data that support the findings of this study are available from the corresponding author upon reasonable request.
