# Supplementary material for: Multi‐Wavelength Achromatic 3D Meta‐holography with Zoom Function
Source: Adv Sci (Weinh). 2025 Apr 25;12(28):2501881. doi: 10.1002/advs.202501881 (PMC12302553; doi:10.1002/advs.202501881)
Supplement: Supplementary file 1 — Supporting Information [file ADVS-12-2501881-s001.docx]

SUPPLEMENTARY MATERIAL

**Multi-wavelength achromatic 3D meta-holography with zoom function**

Chao Liu^1, †^, Yi Zheng^1, †^, Di Wang^1^, Qian Huang^1^, Xiao-Wei Li^1^, Fan-Chuan Lin^1^, You-Ran Zhao^1^, Yi-Wei Zheng^1^, Xiao-Ke Lu^1^, Xin-Ru Li^2^, Xin-Ru Zheng^3^, Xin Xie^3^, Kun Song^3^, Zhen-Fei Li^3^, Wei Lu^2^, Din Ping Tsai^4^, Ruo-Nan Ji^2, 3, *^, and Qiong-Hua Wang^1, *^

*1 School of Instrumentation and Optoelectronic Engineering, Beihang University, Beijing 100191, China.*

*2 State Key Laboratory of Infrared Physics, Shanghai Institute of Technical Physics, Chinese Academy of Sciences, Shanghai 200083, China.*

*^3^ School of Physical Science and Technology, Northwestern Polytechnical University, Xi’an 710129, China.*

*^4^ Department of Electrical Engineering, City University of Hong Kong, Hong Kong 999077, China.*

†*These authors contributed equally to this work.*

**Correspondence: RN Ji, E-mail: jiruonan@mail.sitp.ac.cn;*

*QH Wang, E-mail: qionghua@buaa.edu.cn*

9 pages, 6 figures S1-S6

1. **Generation of the multi-plane 3D meta-hologram**

In the process of generating the 3D meta-hologram, the target 3D object is divided into a series of 2D layers, and the change of the reconstruction depths of different layers can be realized by selecting different transformation orders *a* for the Fractional Fourier transform (FRFT) and inverse Fractional Fourier transform (IFRFT). To verify the feasibility of the multi-plane 3D meta-hologram generation, a 3D object consisting of numbers ‘1’ to ‘9’ located at nine different layers is recorded. The resolution of meta-hologram is 2000×2000. The pixel pitch is set to 0.35 μm, and the transformation orders *a* for the numbers ‘1’ to ‘9’ are set to 0.80, 0.85, 0.90, 0.95 1.00, 1.05, 1.10, 1.15 and 1.20, respectively. The simulation results are shown in Figure S2. It can be seen that the numbers ‘1’ to ‘9’ can be reconstructed accurately. For example, when the number ‘1’ is focused, the blurry states of numbers ‘2’ to ‘9’ increase sequentially, because the difference in transformation order increases.


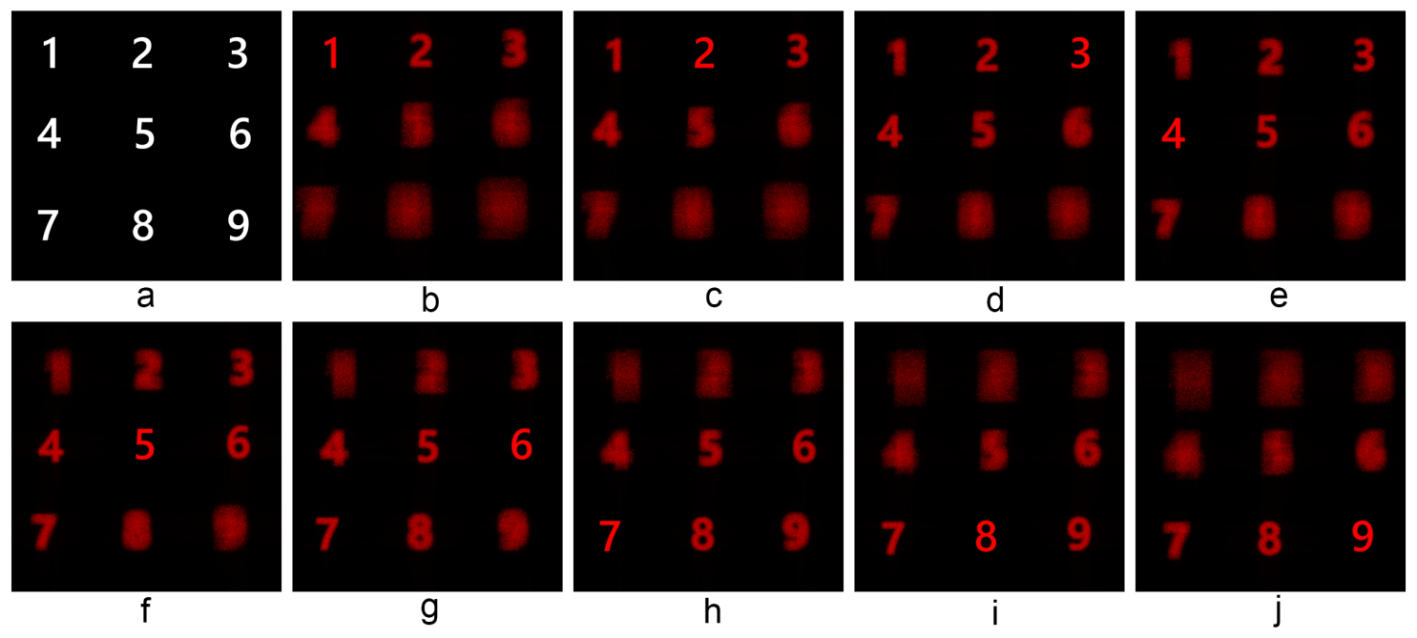


**Figure S1.** Simulation results of the multi-plane 3D meta-hologram. a) Recorded multi-plane object. b-j) Simulation results when numbers ‘1’ to ‘9’ are focused respectively.

1. **Analysis of the zoom range for multi-wavelength achromatic meta-holography**

The zoom range for multi-wavelength achromatic meta-holography is mainly determined by the focal length variation range of the liquid lens and the wavelengths. Considering that the focal length *f* can be expressed in the form of optical power Φ:

$\text{f}=\frac{1}{\Phi}$. (S1)

Thus, according to Equations (3)-(4) in the manuscript, the imaging depth *d* and image size *L* can be expressed as:

$d=\frac{l_{2}\Phi_{1}-1}{l_{2}\Phi_{1}\Phi_{2}-\Phi_{1}-\Phi_{2}}$, (S2)

$L=\frac{m'\lambda d}{m\left(1-l_{2}\Phi_{1}\right)du}$, (S3)

where Φ_1_ and Φ_2_ are the optical powers of liquid lens I and liquid lens II respectively. For a certain reconstruction depth, the size *L_i_* of the meta-holographic image with a specific wavelength *λ_i_* can be expressed as:

$L_{i}=\frac{m'\lambda_{i}}{m\left(\Phi_{i1}+\Phi_{i2}-\Phi_{i1}\Phi_{i2}l_{2}\right)du}\text{,}\begin{matrix} & \Phi_{i1} \end{matrix}\text{,}\Phi_{i2}\in[\Phi_{i\min},\Phi_{i\max}]\wedge\frac{l_{2}\Phi_{i1}-1}{l_{2}\Phi_{i1}\Phi_{i2}-\Phi_{i1}-\Phi_{i2}}=d$. (S4)

The zoom ratio of the meta-holographic image is:

$\text{T}_{i}=\frac{L_{i\max}}{L_{i\min}}$. (S5)

The size variation range of the achromatic meta-holographic image with wavelength *λ*_1,_ ..., *λ_k_* can be expressed as:

$[L_{\min},L_{\max}]=\cap_{i=1}^{k} [L_{i\max},L_{i\min}]$. (S6)

And the zoom ratio of the achromatic holographic image is:

$\text{T}=\frac{L_{\max}}{L_{\min}}$. (S7)

Similarly, the depth variation range of an achromatic meta-holographic image can also be derived. From the above analysis, it can be seen that for a given spectral bandwidth, the chromatic aberration compensation range and zoom range of multi-wavelength meta-holography increase with the enhancement of the zoom capability of the liquid lenses. In addition, since the imaging size variation range is determined by the total focal length variation range of the liquid lens group, the distance setting between the two liquid lenses is important.

In our experiments, when connecting the liquid lenses, the maximum output range of the multi-channel liquid lens driver can cover approximately 0 to 280 mA. Thus, when the two liquid lenses are adjusted synchronously, the zoom range of each liquid lens is approximately -13 D to 13.8 D. When the distance between two liquid lenses is 3 cm, and the illuminating wavelengths are 638 nm, 532 nm and 473 nm, the theoretical size of the meta-holographic image with the letter ‘H’ on the depth plane of 21 cm varies from 3.2 cm to 6.8 cm, 2.7 cm to 5.7 cm, and 2.4 cm to 5.0 cm, respectively, which means that the zoom ratio of the meta-holographic image with each wavelength can reach about 2.1. In addition, when the optical powers of the liquid lenses are both set to 13.8 D, the imaging depth can reach the minimum of 2.7 cm.

1. **Liquid preparation and characteristic test**

Existing liquid lenses still face limitations in zoom range and response time.^[1]^ To break through the limitations, we mainly improve the zoom range and response speed from the perspective of material preparation. Compared with common polymer membrane materials such as ethylene vinyl acetate copolymer, thermoplastic polyurethanes, and polyethylene glycol terephthalate, the polydimethylsiloxane (PDMS) polymer membrane has relatively high elasticity and transmittance. Therefore, the PDMS polymer membrane is selected as a suitable polymer membrane for liquid lens. After preparing the PDMS polymer membrane, the liquid solution is prepared through analysis of multicomponent physicochemical properties. Although the PDMS polymer membrane has many good properties, it may undergo hydrolysis, swelling, or permeation in many types of liquids. According to the principle of similar compatibility, non-polar PDMS polymer membrane may swell in many silicone oil category liquids and aromatic category liquids, resulting in membrane deformation. For aqueous solutions, on one hand, high volatility and narrow liquid phase temperature range may cause the decrease of the stability of the liquid lens. On the other hand, if the solution exhibits acidity and alkalinity, PDMS polymer may be catalyzed to undergo hydrolysis reactions:

$HO[-(CH_{3})_{2}SiO-]_{n}H+H_{2}O\to HO[-(CH_{3})_{2}SiO-]_{n-1}H+(CH_{3})_{2}Si(OH)_{2}$. (S8)

After analysis, neutral alcohol-based substances with strong polarity are selected as the base liquid to ensure the physicochemical stability between the polymer membrane and the liquid. The prepared alcohol solution needs to meet the following three conditions: (1) High thermal stability and low volatility. The liquid phase temperature range needs to cover at least from -20 ℃ to 60 ℃. (2) Moderate viscosity. The viscosity of the liquid should be moderate, approximately in the range of tens of cP, which ensures relatively fast response speed without introducing violent oscillations. (3) Low density. The density should be low and preferably similar to the membrane with a density of 1.044±0.002 g/cm^3^, which helps to maintain the good anti-gravity interference capability of the liquid lens.

The propane-1,3-diol (also known as trimethylene glycol, TMG) is suitable to be the solution for its high thermal stability, low volatility, moderate viscosity and relatively low density. However, it is also necessary to prepare composite liquid with higher refractive index, and lower density closer to the membrane, as this will further improve the zoom range and anti-gravity interference capability of the liquid lens. Different from many traditional aqueous salt solutions where both the refractive index and density are positively correlated,^[2]^ it is found that adding tetrabutylammonium chloride (TBAC) to TMG can not only increase the refractive index of the liquid solution, but also reduce the density, which helps to prepare relatively ideal composite liquid for the proposed liquid lens, as shown in Figures S3a-b.


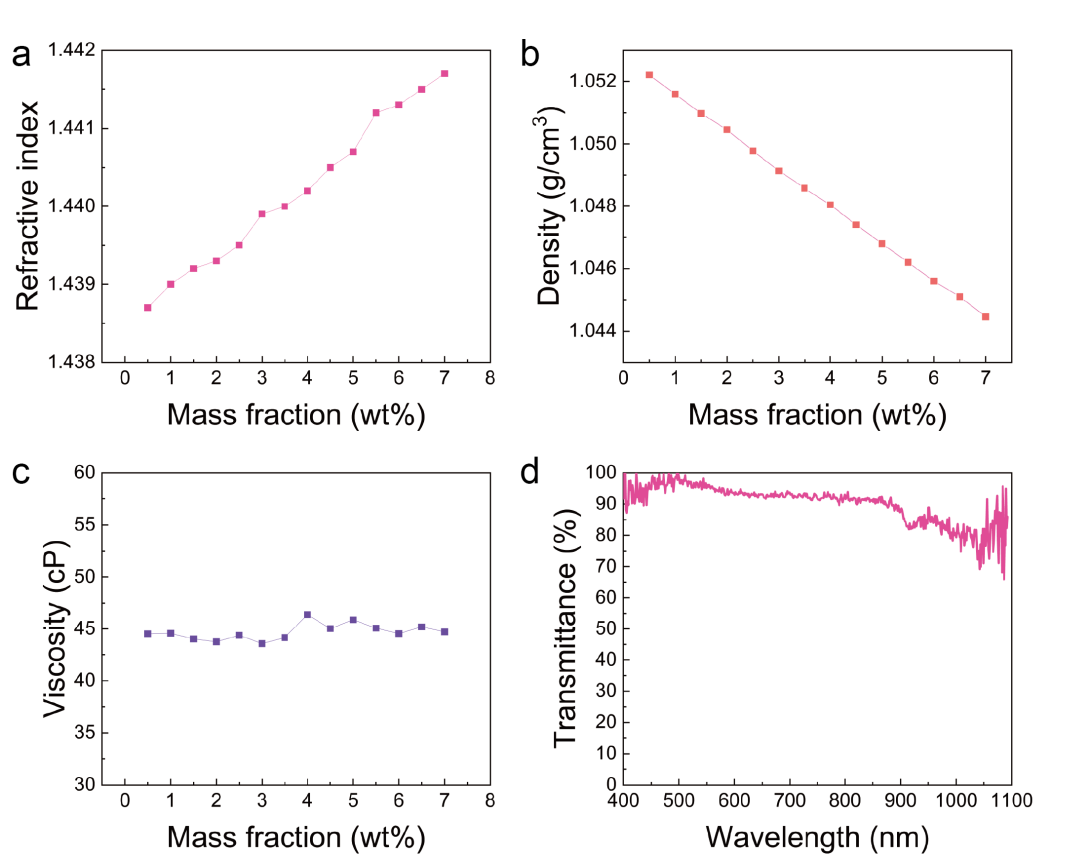


**Figure S2.** Characteristic test results of the liquid. a) Relationship between the density of the liquid and the solute mass fraction. b) Relationship between the refractive index of the liquid and the solute mass fraction. c) Relationship between the viscosity of the liquid and the solute mass fraction. d) Spectral transmittance of the liquid with the mass fraction of 7%.

Meanwhile, we find that as the solute mass fraction increases, the viscosity of the prepared liquid is always within the appropriate range, as shown in Figure S3c. When the mass fraction is approximately 7%, the refractive index of the liquid reaches 1.4407 (under yellow sodium D line), the density is 1.04446 g/cm^3^ which is similar to the membrane, and the viscosity is 44.7 cP. In addition, the transmittance of the liquid is also measured and it is found that the liquid has high transmittance up to at least 87% in the visible light band, and also performs good transmittance characteristics in the near-infrared band, which means that the developed liquid lens has a wide spectral modulation capability, and can be used in multi-wavelength meta-holography with a wide band, as shown in Figure S3d. Such composite liquid with excellent properties lays the foundation for the development of fast tunable and large zoom range liquid lens.

1. **Manufacturing process and performance test of the liquid lens**

The proposed liquid lens is composed of a piece of membrane, the prepared composite liquid, two pieces of window glass, a cavity, a lock ring, a spacing ring, a shell, and a voice coil motor actuator consisting of a group of ring magnet, a coil, a sleeve and two spring plates. The main components of the proposed liquid lens are shown in Figure S5a. The coil is made of copper wire coated with insulating paint on the surface, and is wrapped around the sleeve using dense winding technology. The magnet is magnetized radially, allowing the magnetic field lines to pass through the coil radially. The composite liquid is sealed in the cavity by the elastic membrane and lock ring, forming a flexible optical interface.

When the coil is energized, an axial Ampere force is generated, which drives the sleeve to compress the transparent membrane and composite liquid. Both ends of the sleeve are connected to spring plates. The spring plates can help locate the initial position of the sleeve, which determines the initial optical power of the liquid lens, and can also improve the stability and response speed of the liquid lens to a certain extent. The spacing ring, cavity, lock ring and shell containing upper and lower parts are fabricated by computerized numerical control machining technology. The introduction of reasonable spacing structures and riveting structures improves the integration, tightness and alignment accuracy of the liquid lens, and reduces assembly difficulty. Two pieces of transparent window glass are glued to the bottom of the cavity and the top of the shell respectively for protection and encapsulation. The two poles of the coil are connected to a flexible electrode, and the driving current can be controlled by the self-developed liquid lens driver. The final integrated liquid lens is shown in Figure S5b.

After fabricating the liquid lens, the optical power and response time of the liquid lens are tested. The optical power is directly measured by an optical power meter, and the response time is measured by a self-established setup which consists of a laser, a laser beam expander, the liquid lens to be tested, an aperture and a Si-based amplified photodetector (Type of PDF 10 A/M, Thorlabs, Inc., America). When the focal length of the liquid lens changes under the control of the driving signal, the convergence or divergence state of the light beam passing through the liquid lens varies, so the illuminance received by the photodetector changes accordingly. Based on this principle, the response curve of the liquid lens can be measured by recording the change of the light intensity signal received by the photodetector. It should be pointed out that the photoelectric detector has inherent noise, such as dark current noise, causing fluctuations at the beginning and end of the response curve in Figures 3d-e of the manuscript. In fact, after the liquid lens finishes the response to a step control signal, there is no significant fluctuation of optical power readings when the optical power is tested by the optical power meter with an accuracy of at least 0.1 D, demonstrating the static stability of the liquid lens.

**
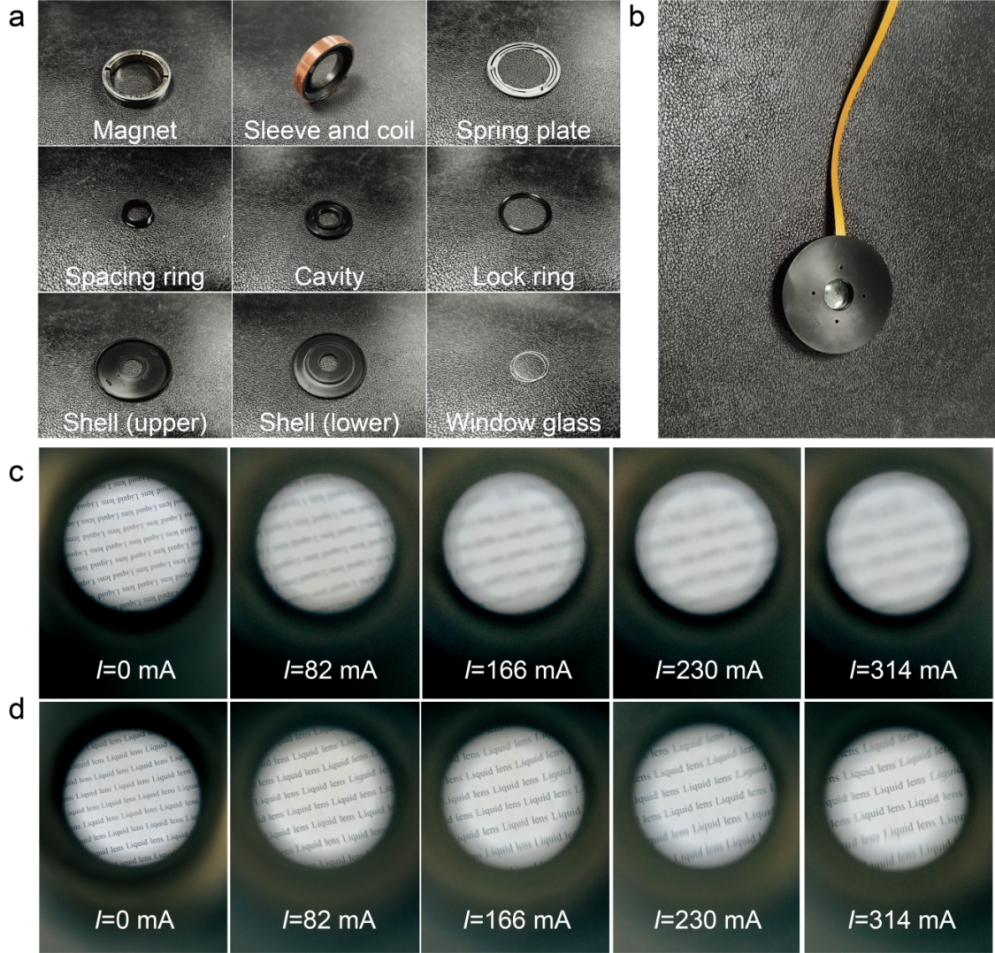
**

**Figure S3.** Fabrication and optical imaging results of the liquid lens. a) Components of the liquid lens. b) Real picture of the liquid lens. c) Images captured by a mobile phone camera without autofocus function when different driving currents are applied to the liquid lens. d) Images captured by a mobile phone camera with autofocus function when different driving currents are applied to the liquid lens.

In addition, we also test the optical imaging performance of the liquid lens. The liquid lens is positioned at the center of the imaging field of view of a mobile phone camera, and a piece of paper with the letters ‘Liquid lens’ written on it is used as the target. To test the maximum zoom range of liquid lenses, the electrode of the liquid lens is connected to a direct-current power source. When the currents from 0 to 314 mA are applied to the liquid lens, the zoom imaging function can be significantly observed. Figures S5c-d show images captured by the mobile phone camera without autofocus function and with autofocus function when different currents are applied to the liquid lens, respectively. It can be seen that the liquid lens has clear zoom imaging effect, which lays the foundation of the multi-wavelength achromatic 3D meta-holography with zoom function.

1. **Design of the multi-channel liquid lens driver**

The zoom function of the meta-holography is achieved through synchronous control of the two liquid lenses. Instead of using two direct-current power sources, we develop an integrated multi-channel liquid lens driver to provide synchronous control driving currents for the two liquid lenses, rather than using two direct-current power sources. The liquid lens controller adopts STM32F103RB as the main control chip which controls the 12-bit high-precision current source chip to output current required for the liquid lenses through the I2C interface. The current output range of the liquid lens driver during no-load operation is approximately 0 to 300 mA, and when the liquid lens driver is connected to the liquid lenses, the maximum output range is tested as approximately 0 to 280 mA. A serial control architecture is adopted for output control of different channels, which has low cost and can be easily achieved. The control time difference for two channels can be controlled within 30 μs, which can be nearly ignored in our proposed method. The upper computer software is developed to conveniently send control signals to the serial port to adjust the output current. Although the loaded output current is slightly lower than the maximum operating current of the liquid lens, the integrated multi-channel liquid lens driver provides more scalability, such as programming control and wireless control.

1. **Transmittance test of the metasurface**

The transmittance test result of the metasurface is shown in Figure S4. In the experiment of multi-wavelength achromatic 3D meta-holographic reconstruction with zoom function, the wavelengths of the lasers mainly involve 450 nm, 473 nm, 520 nm, 532 nm, 638 nm, and 671 nm, and the corresponding transmittances are tested as 53.5%, 43.7%, 30.2%, 32.6%, 56.9%, and 78.0% respectively. It can also be seen that the transmittance of the metasurface between 400 nm and 1100 nm is more than 26%, indicating that the metasurface operates with a very wide spectral response band. Owing to the wavelength-independent phase modulation mode of the metasurface based on Pancharatnam-Berry geometric phase, meta-holographic images with any wavelengths can be generated by illuminating the metasurface with light of corresponding wavelength within the spectral response band as long as the transmittance is non-zero.


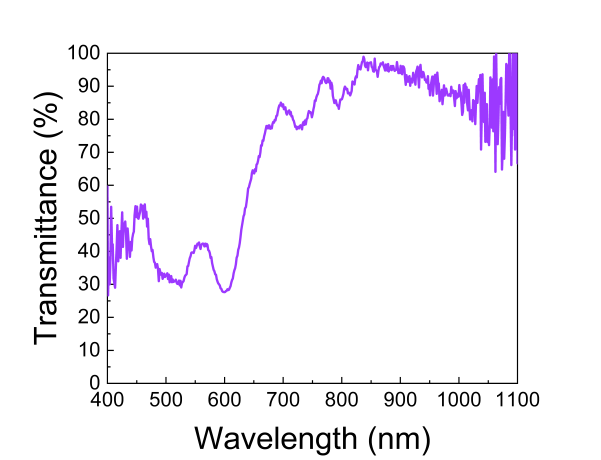


**Figure S4.** Transmittance test result of the metasurface.

1. **Zoom ratio of the multi-wavelength meta-hologram**

When the receiving screen is placed at a specific depth, the size of the meta-holographic image can be adjusted by controlling the driving currents of the liquid lenses. For the meta-holographic image with a certain wavelength, there is a corresponding zoom range. In the experiment of multi-wavelength achromatic 3D meta-holographic reconstruction with zoom function, three lasers with wavelengths of 638 nm, 532 nm and 473 nm respectively are selected as illumination sources. When the metasurface is illuminated with the three lasers respectively, the size variation range of the meta-holographic images of the letter ‘H’ at the depth of 21 cm can cover 3.4 cm to 6.5 cm, 2.7 cm to 5.4 cm, and 2.4 cm to 4.6 cm, respectively, as shown in Figure S6. The zoom ratios of meta-holographic images with different wavelengths are generally consistent, and the slight differences are due to the dispersion of liquid lenses. Especially at the boundary of the selected spectral band, the zoom ratio may slightly decrease. From the results, it can also be seen that the achromatic zoom range is actually the intersection of the zoom ranges of meta-holographic images with different wavelengths, which covers 3.4 cm to 4.6 cm in the validation experiment.

To achieve chromatic aberration compensation in color meta-holography with a wider band, it is necessary to explore some methods to further expand the zoom range of the meta-holography. One possible method is to increase the zoom range of the liquid lens, specifically by developing liquid materials with a higher refractive index, improving the driving force and displacement range of the voice coil motor, and so on. Another possible method is to increase the number of liquid lenses used in the zoom lens group, but this may lead to a trade-off between zoom performance and spatial size requirement. In addition, it is also possible to improve the zoom range and optimize the zoom lens group by combining some advanced devices and technologies.^[3]^

**
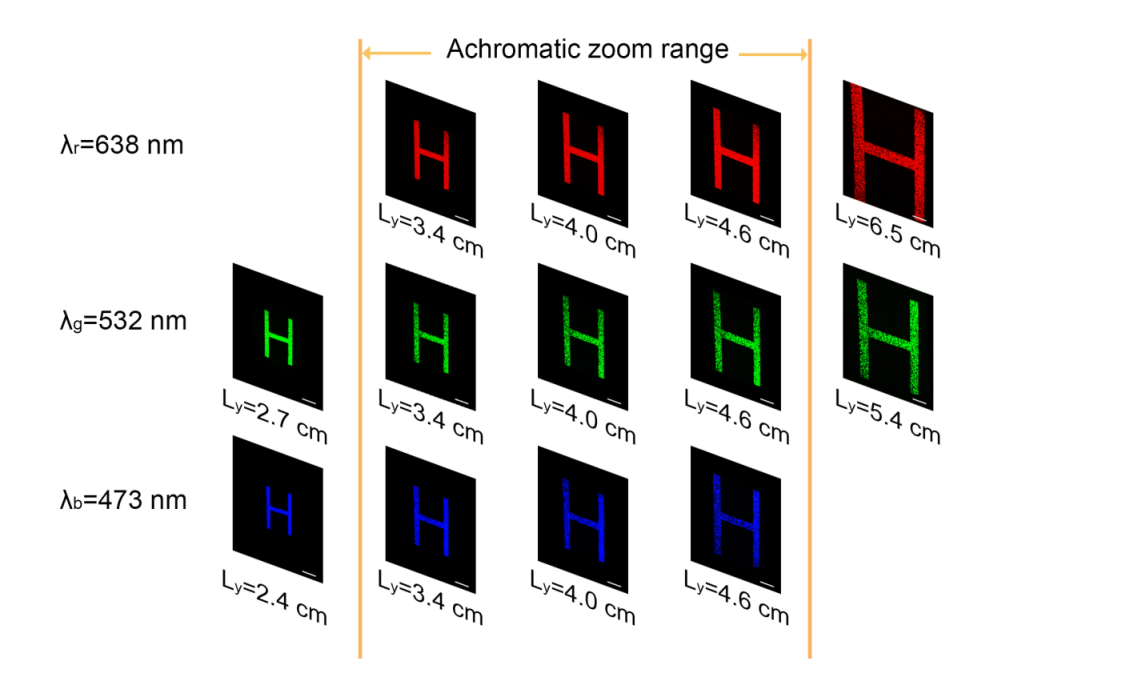
**

**Figure S5.** Zoom results of the multi-wavelength meta-holography with wavelengths of 638 nm, 532 nm, and 473 nm. (All scale bars in the bottom right corner of the pictures represent 1 cm.)

1. **Meta-holographic reconstruction using other wavelengths**

Both the developed metasurface and liquid lenses have wide spectral bands. Meanwhile, chromatic aberration can also be well compensated. To further validate the universality of our proposed method, we additionally select three lasers with different wavelengths of 671 nm, 520 nm and 450 nm as the illumination sources. When the metasurface is illuminated with the three lasers respectively, the size variation ranges of the meta-holographic images of the letter ‘H’ at the depth of 21 cm can cover 3.6 cm to 6.8 cm, 2.6 cm to 5.2 cm, and 2.3 cm to 4.4 cm, respectively, as shown in Figure S6. Therefore, by adjusting the driving currents of the liquid lenses, the achromatic meta-holographic images with sizes between 3.6 cm and 4.4 cm can be reconstructed. Compared with traditional methods which can only compensate chromatic aberration for several specific wavelengths, our proposed method enables zoom modulation and chromatic aberration compensation for meta-holograms with any wavelengths.

**
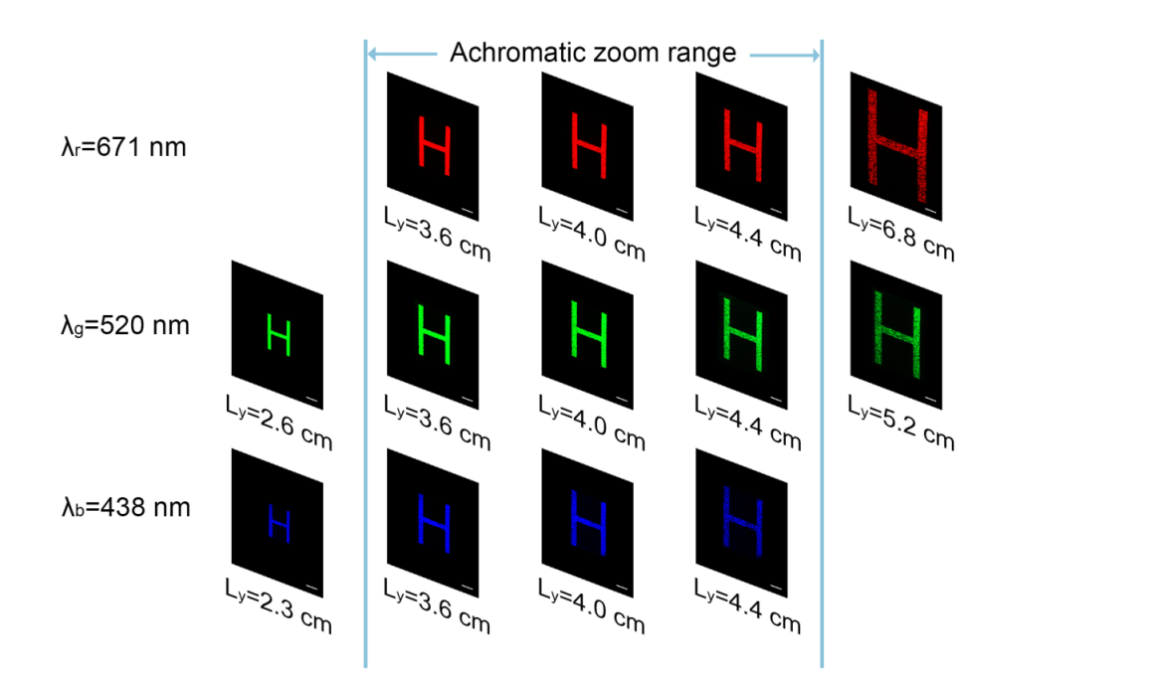
**

**Figure S6.** Reconstruction results with wavelengths of 671 nm, 520 nm, and 438 nm. (All scale bars in the bottom right corner of the pictures represent 1 cm.)

**References**

1. C. Liu, Y. Zheng, R. Yuan, Z. Jiang, J. Xu, Y. Zhao, X. Wang, X. Li, Y. Xing, Q. Wang. Tunable liquid lenses: emerging technologies and future perspectives. *Laser Photonics Rev.* **2023**, *17*, 2300274.
2. N. An, B. Zhuang, M. Li, Y. Lu, Z.-G. Wang. Combined theoretical and experimental study of refractive indices of water–acetonitrile–salt systems. *J. Phys. Chem. B* **2015**, *119*, 10701.
3. Y. Chia, W. Liao, S. Vyas, C. H. Chu, T. Yamaguchi, X. Liu, T. Tanaka, Y. Huang, M. K. Chen, W. Chen, D. P. Tsai, Y. Luo. In vivo intelligent fluorescence endo-microscopy by varifocal meta-device and deep learning. *Adv. Sci.* **2024**, *11*, 2307837.
